# Prevalence and Determinants of Current Smoking and Intention to Smoke among Secondary School Students: A Cross-Sectional Survey among Han and Tujia Nationalities in China

**DOI:** 10.3390/ijerph14111323

**Published:** 2017-10-30

**Authors:** Xianglong Xu, Dengyuan Liu, Manoj Sharma, Yong Zhao

**Affiliations:** 1 School of Public Health and Management, Chongqing Medical University, Chongqing 400016, China; xianglong1989@126.com (X.X.); dengyuanliu@foxmail.com (D.L.); 2 Research Center for Medicine and Social Development, Chongqing Medical University, Chongqing 400016, China; 3 Collaborative Innovation Center of Social Risks Governance in Health, Chongqing Medical University, Chongqing 400016, China; 4 Department of Behavioral and Environmental Health, Jackson State University, Jackson, MS 39213, USA; manoj.sharma@jsums.edu

**Keywords:** smoking, intention to smoke, frequency of smoking, Han nationalities, Tujia nationalities, secondary school students, China

## Abstract

*Objectives:* This study examined the patterns and determinants of current smoking and intention to smoke among secondary school students of Han and Tujia nationalities in China. *Methods:* A cross-sectional survey was conducted in three regions, namely, Chongqing, Liaocheng, and Tianjin, of China in 2015. A structured self-administered questionnaire was used for data collection. *Results:* Of the total subjects (*n* = 1805), 78.9% were ethnic Han and 21.1% were ethnic Tujia. Overall 9.4% (Han: 7.7%; Tujia: 15.5%) secondary school students were smokers and 37.28% smoked more than once per day. Of the non-smoker students (*n* = 1636), 17.4% have an intention to smoke. A total of 81.1% of students reportedly had never been taught throughout school about smoking or tobacco prevention. When compared to the students who were taught in the school about smoking or tobacco prevention (18.90%) students who were never taught were more likely to smoke (OR = 2.39; 95% CI = 1.14–5.01). As compared to Han nationality students who were from Tujia nationality were more likely to smoke (OR = 2.76; 95% CI = 1.88–4.04) and were more likely to have a higher frequency of smoking (95% CI (0.88, 0.88), *p* = 0.010). Non-smokers who were high school students (OR = 4.29; 95% CI = 2.12–8.66), whose academic performance were situated in the last 25% (OR = 2.23; 95% CI = 1.48–3.34) and lower than 50% (OR = 1.50; 95% CI = 1.02–2.20) were more likely to have an intention of smoking. *Conclusions:* About one in ten secondary school students was a smoker, one in three smokers smoked more than one time per day, and a quarter of non-smokers had an intention of smoking in China. Smoking rate was higher among students from Tujia than the Han nationality. This study provided some important information for future tobacco control programs among secondary school students in the ethnic minority autonomous region and minority settlements in a multi-ethnic country.

## 1. Background

Tobacco use can increase the risk of many diseases and is becoming a global problem and smoking among teens is becoming a serious public health problem. Smoking can increase the risk of respiratory diseases, cardiovascular diseases, and many different forms of cancer [1]. Prevention of tobacco use among the youth is critical to ending the tobacco use epidemic that starts primarily in adolescence [2]. More than 300 million Chinese smoke, and about one million people die from tobacco-related diseases each year [3]. About 30.5% of male secondary school students in an urban Chongqing city of China were smokers [4]. About 25.6% of secondary school students in Sichuan province of China have smoking experience [5]. According to 2014 China Youth Tobacco Report, nearly 7% of middle school students were using tobacco products, and of which, 19.9% of middle school students had tried tobacco products [6]. Approximately 90% of adult smokers initiated smoking before 18 years of age in United States [2], and approximately 40% of adolescent smokers started smoking before 10 years of age in China [7]. Once adolescents begin to smoke, they are likely to become regular smokers and the earlier one starts smoking the higher is the risk of addiction in later life [8].

China is a multi-ethnic country with 56 ethnic groups, including five ethnic minority autonomous regions, and the culture of smoking is common. The Han population is the world’s most populous ethnic group. According to the sixth national Census in China in 2010, the Tujia nationality, with a population of about 8,353,912, ranked 7.34% among the 55 minorities of China [9,10]. The Tujia nationality was the largest minority population in Chongqing with a population of about 1.398 million [9]. *Guanxi* (i.e., relationships and connections), *renqing* (i.e., feelings and social favors), and *mianzi* (i.e., face and respect), are all dominant Chinese values among the Han population that give meaning to the gift-giving behavior and its role in maintaining harmonious social bonds and interaction [11]. Gifting and sharing cigarettes is also common among the Tujians. Most Tujia families have very smooth and fertile tobacco lands where they plant every year. Tujians often smoke after work. Gifts are instruments for establishing and maintaining interpersonal relationships in Chinese culture [12]. Giving cigarettes as gifts is common in weddings and funerals, when building houses, and on birthdays. Tujia persons bring tobacco to give as gifts in these events. When Tujians meet acquaintances, regardless of the position and wealth, they bring cigarettes. 

Many factors are responsible for a young person to initiate and sustain the habit of cigarette smoking. Individual factors including attitudes such as smoking intention [13], sociodemographic factors including gender [14], race [15], academic problems and grade point average [16] are associated with students’ smoking behaviors. A previous study also found that the smoking rate of the Tujia nationality is significantly higher than that of Han nationality [17]. In China, the school-based smoking intervention in middle school students increased smoking-related knowledge in both Tibetan and Han ethnic groups but the intervention did not change the prevalence of smoking in both ethnic groups [18]. An Austrian study found that a school-based program was more effective than standard or general anti-smoking education offered by their schools [19].

Understanding the determinants of intention to smoke and frequency of smoking is an important issue on the future of tobacco control. Intention usually represents a decision to exert effort to perform the behavior [20]. Since many theories in social and health psychology assume that intention cause behaviors, thus the intention to smoke is considered to be the major predictor of smoking behavior [21,22]. To assess the magnitude and implication of smoking behavior, it’s necessary and important to understand dependence patterns of the higher frequency of smoking among smokers and identify those at a high risk of becoming addicted. There is limited knowledge about the magnitude and implication of smoking among secondary students. A better understanding of the determinants of smoking and intention to smoke among secondary school students is important in tobacco control in China. The aims of proposed research were following:

(1) To probe the prevalence of smoking and intention to smoke among students between Han and Tujia nationalities in China;
(2) To probe the determinants of smoking and frequency of smoking among smoker students between Han and Tujia nationalities in China;
(3) To probe the determinants of intention to smoke among nonsmokers students between Han and Tujia nationalities in China.

## 2. Materials and Methods

### 2.1. Research Methods

#### 2.1.1. Sample Size

According to the literature [5], the prevalence rate of smoking among secondary school students in China is about 25.6%, we set P = 0.256; Q = 1 – P = 1 – 0.256 = 0.744; margin of error $d=0.10\times P=0.10\times 0.256=0.0256,Z_{\alpha }=1.96$.

So sampling size is $N=\frac{Z_{\alpha }^{2}\times pq}{d^{2}}=\frac{1.96^{2}\times 0.256\times 0.744}{0.0256^{2}}=1116$; sampling size = 1116. The actual total sample size was 1813 individuals in the survey.

#### 2.1.2. Population and Sample

The cross-sectional study was conducted among secondary school students in three areas, namely, Chongqing, Liaocheng, and Tianjin, China in 2015. We adopted the proportional sampling method to survey secondary school students in three areas. We selected one secondary school in the Jiulong Po District and one secondary school in ShiZhu County in Chongqing. We selected one secondary school in the DongLi District in Tianjin, one secondary school in GuYang County in Liao Cheng. A convenience sampling of selected classes consisting of students within these schools was invited to participate. The inclusion criteria for student participation in the study were: studying in the 7th to 12th grades in the selected school district in China, the junior middle school ranks from 7th to 9th grade and the senior high school ranks from 10th to 12th grade. A total of 1813 completed questionnaires were collected after distributing 1857 questionnaires. The response rate was 97.6%. Eight responses were deleted because of missing data, resulting in a final sample size of 1805 for the analysis. Written informed consent was obtained from school teachers and verbal informed consent was obtained from the students. This study was approved by the Ethics Committee of the Chongqing Medical University (2016001). 

### 2.2. Questionnaire

#### 2.2.1. Demographic Questions

Sociodemographic data included age, gender (male/female); school category was divided into middle school and high school; nationality was divided into Han population and Tujia population; and, academic performance in class was divided into top 25%, upper 50%, lower 50% and last 25%.

#### 2.2.2. Classes about Smoking or Tobacco Prevention

The numbers of classes have been taught throughout school about smoking or tobacco prevention was divided into never, one class lesson, two class lessons, and three or more class lessons. 

#### 2.2.3. Smoking Status

The survey included one question about smoking status. “Do you smoke? In this survey, it meant smoking of cigarettes, hand-rolled cigarettes, bidis, pipes, hookahs or cigars. Response options were “yes”, and “no”, therefore, smoking status was divided into smokers and non-smokers.

#### 2.2.4. Frequency of Smoking

The frequency of smoking among smokers was divided into “I smoke at least once a day” “ I do not smoke daily, but at least once a week” “I do not smoke weekly, but at least once a month” and “I smoke less than once a month”.

#### 2.2.5. Intention to Smoke

Intention to smoke among non-smoker was divided into “I will never start smoking”, “I might start smoking within the next five years”, “I might start smoking within the next six months”, and “I might start smoking within the next month”.

#### 2.2.6. Questionnaire Interview 

In this cross-sectional study, we selected one secondary school in the Jiulong Po District and one secondary school in ShiZhu County in Chongqing. We selected one secondary school in the DongLi District in Tianjin, one secondary school in GuYang County in Liao Cheng in Shandong province. Tujia nationality students were all from ShiZhu County in Chongqing. Han nationality students were from Chongqing, Liaocheng, and Tianjin. The investigators from Chongqing Medical University were trained. The investigation site was controlled by the investigator. The respondents filled in the questionnaires item-by-item. The questionnaires were recycled after the on-the-spot audit and error correction. The investigation process entailed explaining to the students that anonymity will be maintained, and steps were taken to ensure the authenticity and the reliability of the questionnaire.

### 2.3. Survey Implementation

The pilot test was carried out in a secondary school where 30 students took the test. We modified the questionnaire according to the results of the pilot test, especially on the presentation of questions and further improved the answer options of the questions. The survey was group-administered, and was administered by teachers and research staff members in classrooms. Based on the experience of the pilot study, the students were contacted in their classroom before or after classes by a trained research staff member in the current study. First, the teacher of this class gave a description about this survey. Then, research staff members assured that all of the students had assurance of privacy and explained how to fill in the questionnaire. The questionnaire did not need a signature of one’s name. 

### 2.4. Data Analyses

Participant characteristics were presented with descriptive analysis (means, standard deviations, frequencies and percentages). The χ^2^ test was adopted to compare differences in categorical variables, and *t* test was used to compare the differences in continuous variables in two groups. Logistic regression analysis was performed to predict factors associated with smoking among all participants. Logistic regression analysis was performed to predict factors associated with intention to smoke among non-smokers. Ordered multivariate logistic regression was conducted to predict the factors that were associated with higher frequency of smoking among smokers. All of the statistics were performed using two-sided tests, and statistical significance was considered at *p* < 0.05. All data analyses were performed using statistical software (SAS version 9.1.3; SAS Institute, Cary, NC, USA).

## 3. Result

### 3.1. Characteristics of the Sample

We included 1805 students (males 48.6%, age 16.2 ± 1.3 years), consisted of 1424 (78.89%) Han nationalities and 381 (21.1%) Tujia nationalities and 9.4% were smokers. A total of 1637 (90.7%) participants were middle school students. About 81.1% of students had never been taught throughout school about smoking or tobacco prevention. Among smoked students, 42.0% smoked less than one time per month, 37.3% smoked more than one time per day, 12.4% smoked more than one time per month, and 8.3% smoked more than one time per week. Among non-smoker students, 6.6% thought they intended to smoke in the next month, 1.0% thought they would smoke within next six months, 9.7% thought they would smoke within next five years, and 82.6% thought they would never smoke. Statistically significant differences were observed in “age” (t = −3.42, *p* = 0.0006), “gender” (χ^2^ = 84.2929, *p* < 0.0001), “nationality” (χ^2^ = 21.3334, *p* < 0.0001), “academic performance in class” (χ^2^ = 22.7234, *p* < 0.0001), and “school category” (χ^2^ = 1.0758, *p* = 0.2996) between smokers and non-smokers. The comparison between the school category (χ^2^ = 1.08, *p* = 0.2996), the number of classes have been taught throughout school about smoking or tobacco prevention (χ^2^ = 2.27, *p* = 0.5188) of smokers and non-smokers yielded no statistically significant difference. (Please see Table 1).

### 3.2. Multivariate Logistic Regression Analysis for Factors Associated with Smoking 

Multivariate logistic regression among total populations found that the students who were 18–19 years old (OR = 1.80; 95% CI = 1.15–2.83) were more likely to smoke than students aged 13–17 years old. Tujia ethnicity students (OR = 2.76; 95% CI = 1.88–4.04) were more likely to smoke than Han ethnicity students. Students whose academic performance in class in the last 25% (OR = 2.86; 95% CI = 1.73–4.71) were more likely to smoke than in top 25%. Females (OR = 0.16; 95% CI = 0.11–0.25) were less likely to smoke than males. (Please see Table 2).

Multivariate logistic regression among students of Han nationality, those who were 18–19 years old (OR = 2.24; 95% CI = 1.37–3.65) were more likely to smoke than students aged 13–17 years old. Students whose academic performances in class in the last 25% (OR = 3.18; 95% CI = 1.78 to 5.66) were more likely to smoke than those who were in top 25%. Students who had been taught three or more class lessons on smoking or tobacco prevention (OR = 2.39; 95% CI = 1.14–5.01) were more likely to smoke than those who had never been taught. Females (OR = 0.27; 95% CI = 0.17–0.43) were less likely to smoke than males. Multivariate logistic regression among Tujia ethnicity students, females (OR = 0.04; 95% CI = 0.02–0.11) were less likely to smoke than males (please see Table 2).

### 3.3. Ordered Multivariate Logistic Regression for Factors Associated with Higher Frequency of Smoking among Smokers

Ordered multivariate logistic regression found that students who were 18–19 years old (95% CI (1.80, 0.20), *p* = 0.016) were more likely to have a higher frequency of smoking than students aged 13–17 years old. Students who were Tujia ethnicity (95% CI (1.52, 0.20), *p* = 0.011) were more likely to have a higher frequency of smoking than Han ethnicity. Female students (95% CI (−2.01, −0.39), *p* = 0.004) were less likely to have a higher frequency of smoking than male students (please see Table 3).

### 3.4. Multivariate Logistic Regression Analysis for Factors Associated with Intention to Smoke among Non-Smokers

Multivariate logistic regression analysis found that high school students (OR = 4.29; 95% CI = 2.12–8.66) were more likely to have an intention of smoking than middle school students. Students whose academic performance were in the last 25% (OR = 2.23; 95% CI = 1.48–3.34) and lower than 50% (OR = 1.50; 95% CI = 1.02–2.20) than students were in top 25% were more likely to have an intention of smoking among non-smokers. Female non-smokers (OR = 0.22; 95% CI = 0.17–0.30) were less likely to smoke than male non-smokers (please see Table 4).

## 4. Discussion

A total of 9.36% of secondary school students, including 9.6% of high school students and 7.1% of middle school students were smokers, a slightly higher prevalence rate than 6.9% among Chinese middle school students that were found in another study [6]. It was lower when compared to a previous study with secondary school students in Sichuan province of China, in which smoking experience accounted for 25.6% [5]. The prevalence of smoking was 7.7% and 15.5% among Han students, Tujia students, respectively. Possible reason is that 21.1% of Tujia nationalities students were included in this study and the smoking rate among Tujia nationalities was higher than Han nationalities. Another possible reason is the effect of environmental norm in Sichuan province where cigarette industry contributes highly to family income and community development because of the high tobacco production in this province [23], which may promote a greater acceptance in this region. In this study, students were from Chongqing, Shandong, and Tianjin. The previous study also showed and discussed the importance of external environment [5] and future studies should take related measures to change the local social norm especially among middle school students. About 37.3% of smokers smoked more than one time every day, which is higher than the frequency both in Shanghai of 6.6% [24] and 20.1% [25] among Hong Kong secondary students, but lower than the frequency of 43.7% among Ireland middle school smokers [26]. Most of them may be, or will be, experimental smokers (smoking one or two puffs of a cigarette) and a previous study found that experimental smokers are more likely to develop to regular smokers [27] and the earlier students choose to smoke the higher risk of addiction in later life [8]. Therefore, future tobacco control will be needed among secondary school students in China, especially among Tujia students.

More than eighty secondary students had never been taught throughout school about smoking or tobacco prevention. Currently, in order to pursue high graduation rates, some areas of China’s primary and secondary schools may reduce health education courses. A previous study also indicated that the school-based smoking intervention in middle school students did not change the prevalence of smoking in both ethnic groups in China [18]. One possible reason is that general smoking prevention class offered by schools can provide some knowledge related to smoking [18] but not provide effective tobacco prevention rejection skills and other behavior change specific tobacco prevention methods. Another possible reason is that teachers of these classes may be not professionally trained in health education methods. Students who were taught three or more class lessons on smoking or tobacco prevention were still more likely to smoke among Han nationality. An Austrian study on school-based program found health education class issued by professional institutes was more effective than class offered by schools themselves [19]. It is advisable and effective for health related workers to perform a prevention program both in elementary school and middle or junior school on preventing smoking [28,29]. Chinese government education departments may strengthen the supervision of the secondary school curriculum; for example, strengthen the supervision of health education curriculum in aspects of tobacco control. Furthermore, since Chinese population is so large, it is necessary to adopt cost-effective health education strategies to improve to health education and health literacy of secondary school students, such as making full use of mass media and social media.

Students of the Tujia nationality were more likely to smoke than those of Han nationality. A previous study showed that the smoking rate of Tujia nationality was significantly higher than that of the Han nationality [17]. A previous study also found a highly prevalence of smoking among Tujia nationality middle students in China, especially the male middle students [30]. An American research found that minority ethnic groups were significantly different from Caucasians [31]. Possible reason is that students of Tujia nationality were not cared and well supervised because numerous Tujia parents work in other regions and those students were mainly cared by their grandparents [32]. Another reason is the influence of peer pressure including classmates and friends [33], especially for those whose friends dropped out of school or contacted with social personnel closely who had greater possibility to smoke [34]. Additionally, most populations of Tujia nationality have lived together in southeast Chongqing with a relatively lower socioeconomic status (SES). Previous studies emphasized the importance of SES on smoking that those whose SES was in a lower level were more likely to smoke [35,36]. A Chinese research also showed that prevalence of smoking in southeast Chongqing was higher than in other regions of Chongqing [37]. This study further indicated the difference of smoking in two nationalities. This study provides more detailed consideration of preventing teenagers from smoking, especially paying more attention to specific subgroup (Tujia nationality). Future studies should pay greater attention to students to help them quit smoking and process skills of injecting tobacco. 

Students aged 18–19 years old were more likely to smoke than those aged 13–17 years old in Han nationality, but not in the Tujia nationality. It is consistent with a previous Chinese study that a higher age group had a higher possibility to smoke [38,39]. Tujia nationality students aged 18–19 years old does not contribute to a higher rate of smoking than students aged 13–17 years old. Possible reason is that middle school students of Tujia ethnicity were more likely to become an experiment smoker under the stronger smoking culture [23] and less stricter family education generally. A previous Chinese study focusing on students of Tujia ethnicity also found the phenomenon that 43.72% male Tujia ethnicity students and 15.59% female Tujia ethnicity students who were less than 13 years old were experiment smokers [30]. It may indicate that smoking control interventions should be taken earlier for Tujia ethnicity students. However, future studies are needed to verify the association between age and smoking among Tujia nationality.

Smokers aged 18–19 years old were more likely to have a higher frequency of smoking. It is similar with a previous study that students of higher age had a higher smoking frequency [26]. Although students with higher age had a higher knowledge level on hazard of smoking initiation [27], they had a higher smoking frequency. Future studies should focus on those students with a higher age, and interventions should also be taken for students of a lower age to prevent their experimental smoking behavior. It is beneficial to take related prevention or intervention on smoking early to stop them to be an experimental smoker or develop a regular smoker [28]. A study also showed the best age to perform smoking prevention was around 15 years of age [27]. Students who were of Tujia nationality were more likely to have a higher frequency of smoking. A previous study also found that students of Tujia nationality had other unhealthy behaviors, such as, drinking behavior, a lack of physical activity, and unhealthy diet [30]. The occurrence of unhealthy behaviors including higher smoking frequency may be linked to the lower SES. Thus, future studies and health education should focus on smokers who aged 18–19 years old, were Tujia nationality to reduce the high frequency of smoking.

Students whose academic performance was in last 25% were more likely to smoke both in Han nationality, but not in Tujia nationality. It’s similar with a previous finding that the level of academic performance was found to be inversely proportional to smoking behavior [40]. Possible reason is that those whose academic performance was lower were more rebellious, depressed, abandoned or pessimistic about their education and career prospects than their students who were outstanding in academic performance and they may smoke to reduce the influence of these negative emotions [38]. However, no significant association was observed between last 25% academic performance and smoking prevalence among Tujia nationality. In future tobacco control, teachers should pay more attention to poor academic performance students among Han nationality. 

Students whose academic performance in class was in the last quartile were more likely to have an intention to smoke. This study further confirms academic problems and grade point average affects smoking in Chinese secondary school students [16]. A study in the Netherlands also showed strong links between the health behaviors and academic achievements of adolescents [41]. Future health education workers and health related workers should pay more attention to those non-smokers with poorer academic performance. Interventions and supervisions among those non-smokers with poorer academic performance should be taken both in school and family to reduce the prevalence of smoking.

High school students were more likely to have an intention to smoke as compared to middle school students. This may further explain the higher prevalence of smoking among higher aged students. A possible reason is that high school non-smokers were more likely to be affected by environment influence including parents, relative, friends, society, and themselves. Previous study also showed key importance of smoking environment, such as parents and friends smoking, media tobacco advertising, and normative expectations affecting the intention to use smoke [42]. The latest Advertisement Law of the People’s Republic of China had banned from setting up tobacco advertisements around the school [43] and local regulations, for example, Beijing Smoking Control Ordinance [44], also forbids selling cigarettes within 100 meters of schools. However, nearly 50% of tobacco sales still have tobacco advertisements [45], and about 25% schools can buy cigarettes within one hundred meters, especially through small retail stores [46]. Hence, future studies should focus on both school and retail stores to decrease the prevalence of smoking. Increasing the frequency of supervision in high schools can be an effective method. 

This study had some limitations. First, there may be a selection bias and the sample was not nationally representative. The sample consisted of pregnant women in three regions, namely three areas, namely, Chongqing, Liaocheng, and Tianjin, China. One school was selected in each selected survey site, not all of the students were included. Second, environmental factors were not included in this study. Some of the environmental influences include the advertising from the tobacco industry [47], access to cigarettes [48], peer pressure, and social norms [49] that are associated with smoking were not included in this study. Third, only 9.31% of participants were high school students, whereas 90.69% of participants were middle school students. The low percentage of high school participants may affect their representativeness, so the study results should be interpreted with caution. Fourth, our study only included secondary students in the urban area and no rural secondary students were included. Smoking prevalence is higher among rural secondary students than among students in the urban China [6]. Replicating our work in rural secondary students would be helpful. Fifth, this study was conducted in China. However, education differs in varying countries because of the different social systems and cultures. Further studies should be conducted in other countries or regions to confirm and improve the applicability of our findings. Sixth, the self-reported data were subject to potential recall bias. Finally, the design of this study was cross-sectional, and such data reduced the ability to make direct causal inferences, explore whether unmeasured factors may better explain the observed relationships we observed, and determine the direction of causality.

## 5. Conclusions

About one in ten secondary school students was a smoker, one in three smokers smoked more than one time per day, and a quarter of non-smokers had an intention of smoking in China. A total of eighty percent of secondary school students had never been taught throughout school about smoking or tobacco prevention. Tujia nationality students were more likely to smoke and have a higher frequency of smoking. This research holds significance for researchers and practitioners in the fields of health promotion and education, which are specific to secondary school students in a multi-ethnic country, and contributes to a deeper understanding of smoking. 

## Figures and Tables

**Table 1:** Characteristics of the participants stratified by smoking status in China (*n*, %).

Variable	Smoker (*n* = 169)	Non-Smoker (*n* = 1636)	Statistical Test
**Age (mean, SD)**	16.5 ± 1.3	16.1 ± 1.3	*t* = −3.42, *p* = 0.0006
**Age**			
13–17 years old	138 (8.8)	1434 (91.2)	
18–19 years old	31 (13.3)	202 (86.7)	
**Gender**			χ^2^ = 84.2929, *p* < 0.0001
Male	139 (15.8)	739 (84.2)	
Female	30 (3.2)	897 (96.8)	
**School category**			χ^2^ = 1.0758, *p* = 0.2996
Middle school students	12 (7.1)	156 (92.9)	
High school students	157 (9.6)	1480 (90.4)	
**Ethnic category**			χ^2^ = 21.3334, *p* < 0.0001
Han population	110 (7.7)	1314 (92.3)	
Tujia population	59 (15.5)	322 (84.5)	
**Academic performance in class**			χ^2^ = 22.7234, *p* < 0.0001
Top 25%	31 (6.7)	431 (93.3)	
Upper 50%	45 (7.6)	544 (92.4)	
Lower 50%	44 (9.7)	408 (90.3)	
Last 25%	49 (16.2)	253 (83.8)	
**The number of classes have been taught throughout school about smoking or tobacco control**			χ^2^ = 2.2674, *p* = 0.5188
Never	131 (9.0)	1333 (91.0)	
One class lesson	16 (10.5)	136 (89.7)	
Two class lessons	3 (8.3)	33 (91.7)	
Three or more class lessons	19 (12.4)	134 (87.6)	

**Table 2:** Logistic regression model for identifying factors that affect smoking among secondary school students stratified by nationality in China.

Effect	Total Populations			Han Ethnicity			Tujia Ethnicity		
	OR ^a^	95% CI	*p*-Value	OR ^b^	95% CI	*p*-Value	OR ^c^	95% CI	*p*-Value
**Age**									
18–19 years old vs. 13–17 years old	1.80	1.15–2.83	0.010	2.24	1.37–3.65	0.001	0.63	0.19–2.09	0.446
**Gender**									
Female vs. Male	0.16	0.11–0.25	<0.0001	0.27	0.17–0.43	<0.0001	0.04	0.02–0.11	<0.0001
**School category**									
High school student vs. Middle school student	1.12	0.59–2.13	0.736	1.11	0.56–2.18	0.772	1.20	0.11–12.74	0.883
**Ethnicity**									
Tujia ethnicity vs. Han ethnicity	2.76	1.88–4.04	<0.0001	-	-	-		-	-
**Academic performance in class**									
Upper 50% vs. Top 25%	1.27	0.78–2.07	0.342	1.31	0.73–2.34	0.360	1.20	0.45–3.15	0.719
Lower 50% vs. Top 25%	1.50	0.91–2.48	0.109	1.22	0.65–2.29	0.535	2.01	0.81–5.01	0.134
Last 25% vs. Top 25%	2.86	1.73–4.71	<0.0001	3.18	1.78–5.66	<0.0001	1.93	0.69–5.42	0.212
One class lesson vs. Never	1.08	0.61–1.93	0.788	1.54	0.79–3.02	0.204	1.20	0.45–3.15	0.719
**The number of classes have been taught throughout school about smoking or tobacco control**									
Two class lessons vs. Never	0.98	0.27–3.55	0.975	2.53	0.69–9.25	0.160	-	-	-
Three or more class lessons vs. Never	1.35	0.76–2.38	0.303	2.39	1.14–5.01	0.021	-	-	-

^a^ Among Total populations, Adjusted for age, gender, school category, nationality, Academic performance in class, The number of classes have been taught throughout school about smoking or tobacco prevention; ^b^ Among Han ethnicity, Adjusted for age, gender, school category, Academic performance in class, The number of classes have been taught throughout school about smoking or tobacco prevention; ^c^ Among Tujia ethnicity, Adjusted for age, gender, school category, Academic performance in class.

**Table 3:** Ordered multivariate logistic regression for factors associated with higher frequency of smoking among smokers in China.

Parameter	Estimate	Standard Error	95% CI	*p*-Value
**Age**				
18–19 years old vs. 13–17 years old	0.98	0.41	1.80, 0.20	0.016
**Gender**				
Female vs. Male	−1.18	0.41	−2.01, −0.39	0.004
**School category**				
High school vs. Middle school	−0.96	0.60	−2.16, 0.20	0.108
Ethnicity				
Tujia ethnicity vs. Han ethnicity	0.85	0.34	1.52, 0.20	0.011
**Academic performance in class**				
Upper 50% vs. Top 25%	0.18	0.45	−0.70, 1.08	0.684
Lower 50% vs. .Top 25%	−0.16	0.46	−1.06, 0.74	0.723
Last 25% vs. Top 25%	0.59	0.46	−0.30, 1.50	0.200

**Table 4:** Logistic regression analysis for factors associated with intention to smoke among non-smokers in China.

Effect	OR ^a^	95% CI	*p*-Value
**Age**			
Age 18–19 years old vs. 13–17 years old	1.26	(0.86, 1.86)	0.238
**Gender**			
Female vs. Male	0.22	(0.17, 0.30)	<0.0001
**School category**			
High school student vs. Middle school student	4.29	(2.12, 8.66)	<0.0001
**Ethnicity**			
Tujia ethnicity vs. Han ethnicity	0.84	(0.58, 1.23)	0.378
**Academic performance in class**			
Upper 50% vs. Top 25%	1.13	(0.78, 1.64)	0.519
Lower 50% vs. Top 25%	1.50	(1.02, 2.20)	0.039
Last 25% vs. Top 25%	2.23	(1.48, 3.34)	0.0001
**The number of classes have been taught throughout school about smoking or tobacco prevention**			
One class lesson vs. Never	0.86	(0.51, 1.45)	0.561
Two class lessons vs. Never	1.02	(0.37, 2.87)	0.965
Three or more class lessons vs. Never	1.02	(0.59, 1.76)	0.959

^a^ Adjusted for age, gender, school category, ethnicity, Academic performance in class, The number of classes have been taught throughout school about smoking or tobacco prevention.
